# Non-immunological enhancement of tumour transplantability in x-irradiated host animals.

**DOI:** 10.1038/bjc.1977.255

**Published:** 1977-12

**Authors:** R. J. Jamasbi, P. Nettesheim

## Abstract

MSC-10 tumour cells (derived from a chemically induced pulmonary squamous-cell carcinoma in DBA/2 mice) were inoculated intramuscularly into thymectomized, X-irradiated isogeneic mice, either 48 h or 6 weeks after thymectomy and X-irradiation. Normal mice and immunologically reconstituted mice served as controls. A marked enhancement in frequency of tumour takes was observed in all groups of animals inoculated with tumour cells 48 h after whole:-body X-irradiation, whether thymectomized, immunologically reconstituted or not. The TD50 decreased to less than 1/10 of that observed in unirradiated controls. When mice were inoculated with tumour cells 6 weeks after X-irradiation, the incidence of tumour takes was similar to that of unirradiated controls, including the thymectomized-irradiated group, which was still severely immunodeficient as measured by antibody formation and skin graft rejection. The experiments indicate that whole-body X-irradiation creates a condition that favours tumour cell survival or growth. This "permissive state" exists only shortly after X-irradiation and is not correlated with the host's level of immunocompetence.


*Br. J. Cancer* (1977) **36**, 723.

### NON-IMMUNOLOGICAL ENHANCEMENT OF TUMOUR TRANSPLANTABILITY IN X-IRRADIATED HOST ANIMALS

R. J. JAMASBI*† AND P. NETTESHEIM†

*From the *University of Tennessee-Oak Ridge Graduate School of Biomedical Sciences and the †Biology Division, Oak Ridge National Laboratory, Oak Ridge, Tennessee* 37830

Received 13 May 1977 Accepted 20 July 1977

**Summary.—MSC-10 tumour cells (derived from a chemically induced pulmonary squamous-cell carcinoma in DBA/2 mice) were inoculated intramuscularly into thymectomized, X-irradiated isogeneic mice, either 48 h or 6 weeks after thymectomy and X-irradiation. Normal mice and immunologically reconstituted mice served as controls. A marked enhancement in frequency of tumour takes was observed in all groups of animals inoculated with tumour cells 48 h after whole-body X-irradiation, whether thymectomized, immunologically reconstituted or not. The $TD_{50}$ decreased to less than 1/10 of that observed in unirradiated controls. When mice were inoculated with tumour cells 6 weeks after X-irradiation, the incidence of tumour takes was similar to that of unirradiated controls, including the thymectomized-irradiated group, which was still severely immunodeficient as measured by antibody formation and skin graft rejection. The experiments indicate that whole-body X-irradiation creates a condition that favours tumour cell survival or growth. This "permissive state" exists only shortly after X-irradiation and is *not* correlated with the host's level of immunocompetence.**

IN RECENT work (Jamasbi and Nettesheim, 1977) we describe the immunological characteristics of a transplantable pulmonary squamous-cell carcinoma (MSC-10) originally induced in a DBA/2 mouse with 3-methylcholanthrene. We found it impossible to induce any detectable degree of transplantation immunity with a variety of immunization procedures (*e.g.*, repeated immunization with X-irradiated tumour cells and/or by transplantation-excision method) and concluded that this tumour was non-immunogenic (which is not to say that the tumour could not have antigens associated with it).

The original purpose of the studies presented here was to determine whether it was possible to detect any subtle immune reactivity against the tumour by comparing the frequency of tumour takes and growth rates in normal and thymectomized, X-irradiated, immunologically suppressed mice. Our studies yielded no indication of an immunological host anti-tumour response. Instead they produced strong evidence that frequency of tumour take (probably due to increased survival of tumour cells) is markedly enhanced in whole-body X-irradiated recipients, and that this enhancement is *not* mediated by an immunological mechanism. Similar findings and conclusions were recently reported by Peters (1975) with a spontaneous adenocarcinoma derived from a CBA mouse. We feel that this enhancing effect of X-irradiation on survival of tumour cells, if it also occurs in humans, might have to be considered when weighing the relative risks and benefits of X-irradiation in cancer therapy.

Address for correspondence: Dr. Paul Nettesheim, Biology Division, Oak Ridge National Laboratory, P.O. Box Y, Oak Ridge, Tennessee 37830 (USA).

### MATERIALS AND METHODS

*Mice.*—We used inbred male DBA/2 (H-$2^d$) mice 8–10 weeks old at the start of the experiment. The animals were maintained in filter-top cages with free access to food and water.

*Tumour.*—The mouse squamous-cell carcinoma (MSC-10) used was originally induced in a male DBA/2 mouse by intra-tracheal injection of 3-methylcholanthrene (Nettesheim and Hammons, 1971). The tumour line has been maintained by serial passages in the strain of origin. Some of the *in vivo* characteristics of this tumour line were reported recently (Williams and Nettesheim, 1973). Frozen pools of tumour-cell suspensions from the 10th *in vivo* passage were used throughout our studies.

*Tumour-cell inoculation.*—After thawing, the tumour cells were spun down and resuspended in Hanks' solution containing 5% foetal calf serum (FCS). Viability was determined by trypan-blue exclusion. Cell counts were made with a haemacytometer, and the cell suspensions were adjusted to the desired concentrations.

After inoculation of $10^4$ or $10^3$ tumour cells into the thigh muscle, mice were inspected for tumour development every other day from Day 10 on. Incidence of tumour take, tumour growth rate and mortality were recorded for a 70- to 100-day period.

*Thymectomy.*—Adult thymectomy was done on 8- to 10-week-old mice according to the technique described by Gross (1959). At the end of the experiments the mice were autopsied, and the absence of thymic tissue was ascertained by gross and microscopic inspection. Controls were sham-thymectomized.

*Immunological reconstitution.*— Whole thymuses from 3-week-old syngeneic donors were implanted i.p. within 24 h after whole-body X-irradiation (one thymus per recipient).

For preparation of spleen-cell suspensions, spleens from syngeneic donors were aseptically removed. The spleen-cell donors were 8–10 weeks old. Single-cell suspensions were prepared and were passed through sterile 200-mesh stainless steel sieves. The cells were washed in Hanks' balanced salt solution with 10% FCS added. Each mouse received $2 \times 10^8$ viable spleen cells i.p. within 24 h after irradiation.

*X-irradiation.*—Normal or thymectomized mice (2 weeks after removal of thymus) were exposed to 600 rad whole-body X-irradiation. Exposures were administered with a 300kVp X-ray unit (GE Maxitron 300) operated at 20 mA, with an added filtration of 3 mm Al and a target-to-object distance of 60 cm. The exposure rate averaged 180 rad/min. The animals were irradiated in a perforated Lucite container attached to a revolving turntable.

*Measurement of humoral and cellular immunity.*—Mice were injected i.p. with $10^8$ sheep red blood cells (SRBC) to induce antibody production. Animals were bled via the tail vein at 4 and 10 days after SRBC injection, and haemagglutinin titres were determined for each animal by standard tube haemagglutinin tests. To test cellular immunity, DBA/2 mice (H-$2^d$) were grafted with C3H (H-$2^k$) mouse skin according to the method of Billingham and Medawar (1951). Skin from isogeneic donors was used for control grafts.

The animals were inspected daily from Day 8 on, and the condition of the grafts was recorded. The day of complete destruction of the grafts was taken as the time of graft rejection.

### RESULTS

### *Effect of thymectomy and X-irradiation on host resistance to tumour transplantation*

Eight- to 10-week-old mice were thymectomized and given a single dose of 600 rad whole-body X-irradiation 2 weeks later. Sham-thymectomized, unirradiated mice served as controls. Forty-eight hours after X-irradiation, the experimental and control animals were inoculated i.m. with either $10^3$ or $10^4$ live MSC-10 tumour cells.

Results of this experiment are summarized in Fig. 1. In the sham-operated, unirradiated animals, a tumour-cell inoculum of $10^4$ live MSC-10 cells (injected i.m.) produced tumours in only 50% of the animals ($TD_{50}$) whereas the same tumour-cell dose produced 100% tumours in thymectomized, X-irradiated mice. Incidence of mortality followed similar trends (data not shown). The dose of $10^3$ MSC-10 tumour cells (one tenth the $TD_{50}$) failed to produce tumours in any of the sham-treated control animals, but produced

FIG. 1.—Effect of thymectomy and X-irradiation on the frequency of tumour take. Animals were exposed to 600 rad of whole-body X-irradiation 2 weeks after removal of thymus glands. Treated and sham-operated mice were challenged i.m. with either $10^4$ or $10^3$ live MSC-10 tumour cells 48 h after X-irradiation. Thymectomized, X-irradiated group (○), and in the sham-thymectomized, unirradiated group (△) receiving $10^4$ MSC-10 cells. Thymectomized, X-irradiated groups (●), and in the sham-thymectomized, unirradiated group (▲) receiving $10^3$ MSC-10 cells. (20 mice per group.)

tumours in 70% of the thymectomized, X-irradiated mice.

*Effect of immunological reconstitution on host resistance to tumour transplantation.*

Thymectomized, irradiated recipients received 2 × $10^8$ syngeneic spleen cells and thymic implants i.p. within 24 h after X-irradiation. Some of the mice were tested for immunocompetence and others were inoculated with live MSC-10 tumour cells at 48 h after whole-body X-irradiation. The various types of control groups were tested simultaneously.

The results of the tests for immunocompetence are summarized in the upper halves of Tables I and II. Thymectomized, as well as sham-thymectomized, X-irradiated mice showed severe impairment of humoral immune response as measured by the haemagglutinin assay. The skin allograft response was severely suppressed in thymectomized, X-irradiated animals and only slightly suppressed in sham-thymectomized, X-irradiated mice. After reconstitution with spleen cells and thymus, the humoral immune response still appeared to be markedly impaired, whilst the skin allograft survival time was only slightly different from that of normal control animals.

The results of the tumour transplantation studies are summarized in Fig. 2. All three X-irradiated groups showed a marked increase in the incidence of tumour take and mortality, even when the animals were reconstituted with spleen cells and had received thymus grafts. Tumour growth rates (not shown) showed no consistent differences between groups.

TABLE I.—*Effect of Immunological Reconstitution on the Haemagglutinin Response of Thymectomized Mice Receiving* 600 *rad of Whole-body X-irradiation* 48 *h or* 6 *Weeks before SRBC Injections**

| Treatment † | Mean $\log_2$ haemagglutinin titre (± s.e.) Day 4 | Day 10 |
|---|---|---|
| | (48 *h after irradiation*) | |
| Thymectomy, 600 rad | Undetectable | Undetectable |
| Sham thymectomy, 600 rad | Undetectable | Undetectable |
| Thymectomy, 600 rad, reconstituted ‡ | 2·2 ± 0·3 | 3·1 ± 0·7 |
| Sham thymectomy, 0 rad | 6·8 ± 0·4 | 8·6 ± 0·3 |
| | (6 *weeks after irradiation*) | |
| Thymectomy, 600 rad | Undetectable | Undetectable |
| Sham thymectomy, 600 rad | 6·4 ± 0·3 | 7·6 ± 0·3 |
| Thymectomy, 600 rad, reconstituted ‡ | 5·0 ± 0·4 | 6·0 ± 0·6 |
| Sham thymectomy, 0 rad | 8·4 ± 0·3 | 9·8 ± 0·1 |

* $10^8$ SRBC injected i.p. either 48 h or 6 weeks after exposure to whole-body X-irradiation.
† 10 mice per treatment group.
‡ 2 × $10^8$ syngeneic spleen cells and a whole thymic graft within 24 h after X-irradiation.

TABLE II.—*Effect of Immunological Reconstitution on the Skin Allograft (Donor C3H) Response of Thymectomized Mice Receiving* 600 *rad of Whole-body X-irradiation* 48 *h or* 6 *Weeks before Skin Grafting**

| Treatment † | Mean skin-graft survival (days ± s.e.) |
|---|---|
| | (48 *h after irradiation*) |
| Thymectomy, 600 rad | > 60 |
| Sham thymectomy, 600 rad | 16·1 ± 0·5 |
| Thymectomy, 600 rad, reconstituted ‡ | 14·5 ± 0·9 |
| Sham thymectomy, 0 rad | 12·3 ± 0·6 |
| | (6 *weeks after irradiation*) |
| Thymectomy, 600 rad | > 60 |
| Sham thymectomy, 600 rad | 11·2 ± 0·6 |
| Thymectomy, 600 rad, reconstituted ‡ | 12·4 ± 0·6 |
| Sham thymectomy, 0 rad | 11·2 ± 0·6 |

* Grafts performed either 48 h or 6 weeks after exposure to whole-body X-irradiation.
† 10 mice per group.
‡ 2 × $10^8$ syngeneic spleen cells and a whole thymic graft within 24 h after X-irradiation.

In a subsequent study, similarly treated groups of mice were tested for immunocompetence and resistance to tumour transplantation 6 weeks after whole-body

FIG. 2.—Effect of immunological reconstitution on tumour transplantability in thymectomized mice receiving whole-body X-irradiation 48 h before tumour-cell inoculation. Animals were exposed to 600 rad of whole body X-irradiation 2 weeks after removal of thymus glands. Each mouse in the reconstituted group received 2 × $10^8$ syngeneic spleen cells and one thymic graft within 24 h after X-irradiation. Treated and sham-operated controls were challenged i.m. with $10^4$ live MSC-10 tumour cells 48 h after X-irradiation. Thymectomized, X-irradiated group (○); thymectomized, X-irradiated reconstituted group (□); sham-thymectomized, X-irradiated group (⬡); sham-thymectomized, unirradiated group (△). (20 mice per group.)

FIG. 3.—Effect of immunological reconstitution on tumour transplantability of thymectomized mice receiving whole-body X-irradiation 6 weeks before tumour-cell inoculation. Animals were exposed to 600 rad of whole-body X-irradiation 2 weeks after removal of thymus glands. Each mouse in the reconstituted group received 2 × $10^8$ syngeneic spleen cells and one thymic graft within 24 h after X-irradiation. Treated and sham-operated controls were challenged i.m. with $10^4$ live MSC-10 tumour cells 6 weeks after X-irradiation. Thymectomized, X-irradiated group (○); thymectomized, X-irradiated, reconstituted group (□); sham-thymectomized, irradiated group (⬡); sham-thymectomized, unirradiated group (△). (20 mice per group.)

TABLE III.—*Influence of Host Immunological Competence on the Development of Distant Metastases**

| Treatment † | No. of mice with lung metastases | Mean no. of lung tumours/ mouse (± s.e.) | Mean size of nodules ($mm^3$ ± s.e.) |
|---|---|---|---|
| Thymectomy, 600 rad | 10 | 7·1 ± 3·9 | 35 ± 11·0 |
| Sham thymectomy, 0 rad | 12 | 7·3 ± 6·4 | 30 ± 8·7 |

* Thymectomized, X-irradiated mice and sham-operated controls were inoculated i.m. with $2 \times 10^4$ live MSC-10 tumour cells 6 weeks after exposure to 600 rad whole-body X-irradiation. Tumour-bearing legs were removed 4 weeks after inoculations. These mice were killed 4 weeks after surgery and the number and size of lung metastases were determined.
† 20 mice per group.

X-irradiation and inoculation of immunocompetent cells. We chose a 6-week period between X-irradiation-reconstitution and testing to allow time for recovery. The results of this study are summarized in the lower halves of Tables I and II and in Fig. 3. The immunological studies showed partial and complete recovery, respectively, of the haemagglutinin and the homograft-rejection response in all but the thymectomized X-irradiated mice. This latter group showed no signs of immunological recovery. The tumour transplantation studies however, show that all groups of animals have reacquired an almost normal degree of resistance to tumour-cell inoculation, namely that of unirradiated, sham-thymectomized, age-matched controls (see Fig. 3). Only 40–50% of the animals developed tumours. Tumour development was actually slightly retarded in groups that had received X-irradiation 6 weeks earlier.

*Incidence of tumour metastases in immunosuppressed mice*

A total of 20 mice were thymectomized and subsequently exposed to 600 rad of whole-body X-irradiation. Six weeks after X-irradiation, a time when humoral as well as cellular immune competence is still severely suppressed (see Tables I and II,) the above group of animals and age-matched, sham-operated control animals were challenged with $2 \times 10^4$ live tumour cells given i.m. The tumour-bearing legs were removed 4 weeks after inoculation. After a further 4 weeks, all animals were killed. Their lungs were removed and fixed, and the number of metastatic nodules was established in cleared and stained lungs with a dissecting microscope (Yuhas, 1973). The results (Table III) show that the incidence of tumour metastasis is not affected by the immunosuppressed state (no metastases were found in any organs other than the lungs.).

### DISCUSSION

The lung squamous-cell carcinoma (MSC-10) is a highly metastatic malignant tumour displaying no, or very weak, immunogenicity, since it is incapable of producing humoral immunity (determined by indirect immunofluorescent antibody techniques and radioimmunoassays) or inducing tranplantation resistance in syngeneic hosts (demonstrated by transplantation-excision method and Winn neutralization tests; Jamasbi and Nettesheim (1977). This study was concerned with the question of whether immunosuppression induced by thymectomy and whole-body X-irradiation would significantly affect survival and growth of cells from this tumour line in syngeneic hosts. The key findings are as follows:

(a) Animals treated with whole-body X-irradiation (with or without thymectomy) show marked decrease in resistance (or enhanced susceptibility), by a factor of $\sim 10$, to tumour-cell inoculation shortly after X-irradiation.

(b) This altered host status is not abrogated by infusion of immunocompetent cells (which partially restores host immunological competence).

(c) Six weeks after X-irradiation (with or without thymectomy) the animals reacquired a "normal" level of host-resistance to tumour transplantation, regardless of their immunological status (*i.e.*, X-irradiated thymectomized mice still have severely depressed immune functions, as measured by antibody formation and skin-graft rejection, yet their resistance to tumour transplantation is the same as that of controls or of immunologically reconstituted mice).

(d) The incidence of spontaneous tumour metastasis is not different from controls in severely immunosuppressed mice 6 weeks after X-irradiation and thymectomy.

The following tentative conclusions can be drawn from these findings. Even in severe states of humoral and cellular immune suppression, local tumour growth, as well as development of distant metastases, may not be significantly altered from normal if the tumour is not immunogenic or only weakly so. The "natural" host-resistance to tumour transplantation is impaired shortly after exposure to a sublethal dose of X-irradiation. However, at least in the case of poorly immunogenic tumours, this is not a result of X-ray-induced immune suppression, since this decreased resistance vanishes with time, even when the state of immunosuppression persists, as in thymectomized, X-irradiated mice.

Our findings confirm and extend those reported by Peters (1975) obtained with a late-transplant generation of tumour cells derived from a spontaneous adenocarcinoma. He showed that the $TD_{50}$ was markedly reduced in whole-body, X-irradiated mice up to at least 4 days after X-irradiation, but was normal in mice made immunodeficient by thymectomy plus X-irradiation several months prior to inoculation of tumour cells. Laparotomy also reduced the $TD_{50}$, but this effect was less reproducible, less striking, and lasted for only a few hours. Attempts to correlate this enhanced transplantability of tumour cells with increased plasma fibrinogen levels failed. Whether the increased incidence of artificial lung metastases, following irradiation of the lung and subsequent i.v. inoculation of tumour cells (*e.g.*, van den Brenk *et al.*, 1973; Withers and Milas, 1973) is the same or a related phenomenon is not certain. Withers and Milas (1973) interpreted this as an X-ray effect on the capillary bed of the lung.

It is possible that the X-ray enhancement of tumour-cell survival reported here might easily be overlooked in studies with more immunogenic tumours, since the immunosuppressive effect of X-irradiation would tend to overshadow the phenomenon. This may be the reason why this apparent non-immunological effect of X-irradiation has not been reported more frequently.

Whether or not the enhanced survival of tumour cells following X-irradiation, and the enhancement of metastases following surgical trauma (for discussion see Peters, 1975) are related phenomena is presently unclear. However, since cancer patients are commonly subjected to either or both procedures, it seems that further elucidation of the mechanisms involved is highly desirable.

This research was jointly sponsored by the National Cancer Institute and the Energy Research and Development Administration under contract with the Union Carbide Corporation. R.J.J. is a postdoctoral investigator, Carcinogensesis Training Grant No. CA 05296–01 from the National Cancer Institute. We thank Drs Joseph H. Coggin, Jr. and R. A. Griesemer for many stimulating discussions.

### REFERENCES

Billingham, R. E. & Medawar, P. B. (1951) The Technique of Free Skin Grafting in Mammals. *Proc. Soc. exp. Biol. Med.*, **28**, 385.

GROSS, L. (1959) Effect of Thymectomy and Development of Leukemia in C3H Mice Inoculated with Leukemic "Passage" Virus. *Proc. Soc. exp. Biol. Med.*, **100**, 325.

JAMASHBI, R. J. & NETTESHEIM, P. (1977) *Int. J. Cancer.* (in press).

NETTESHEIM, P. & HAMMONS, A. S. (1971) Induction of Squamous Cell Carcinoma in the Respiratory Tract of Mice. *J. natn. Cancer Inst.*, **47**, 697.

PETERS, L. J. (1975) Enhancement of Syngeneic Murine Tumour Transplantability by Whole-body Irradiation: A Non-immunological Phenomenon. *Br. J. Cancer*, **31**, 293.

VAN DEN BRENK, H. A. S., BURCH, W. M., ORTON, C. & SHARPINGTON, C. (1973) Stimulation of Clonogenic Growth of Tumour Cells and Metastases in the Lung by Local X-irradiation. *Br. J. Cancer*, **27**, 291.

WILLIAMS, M. L. & NETTESHEIM, P. (1973) Lung Colony Assay with Squamous Cell Carcinoma Derived from Respiratory Tract of Mice. *J. natn. Cancer Inst.*, **51**, 1513.

WITHERS, R. H. & MILAS, L. (1973) Influence of Pre-irradiation of Lung Development of Artificial Pulmonary Metastases of Fibrosarcoma in Mice. *Cancer Res.*, **33**, 1931.

YUHAS, J. M. (1973) Radiotherapy of Experimental Lung Tumors in the Presence and Absence of a Radioprotective Drug, S-2-(3-aminopropylamina)-ethylphosphorothioic Acid (WR 2721). *J. natn. Cancer Inst.*, **50**, 69.


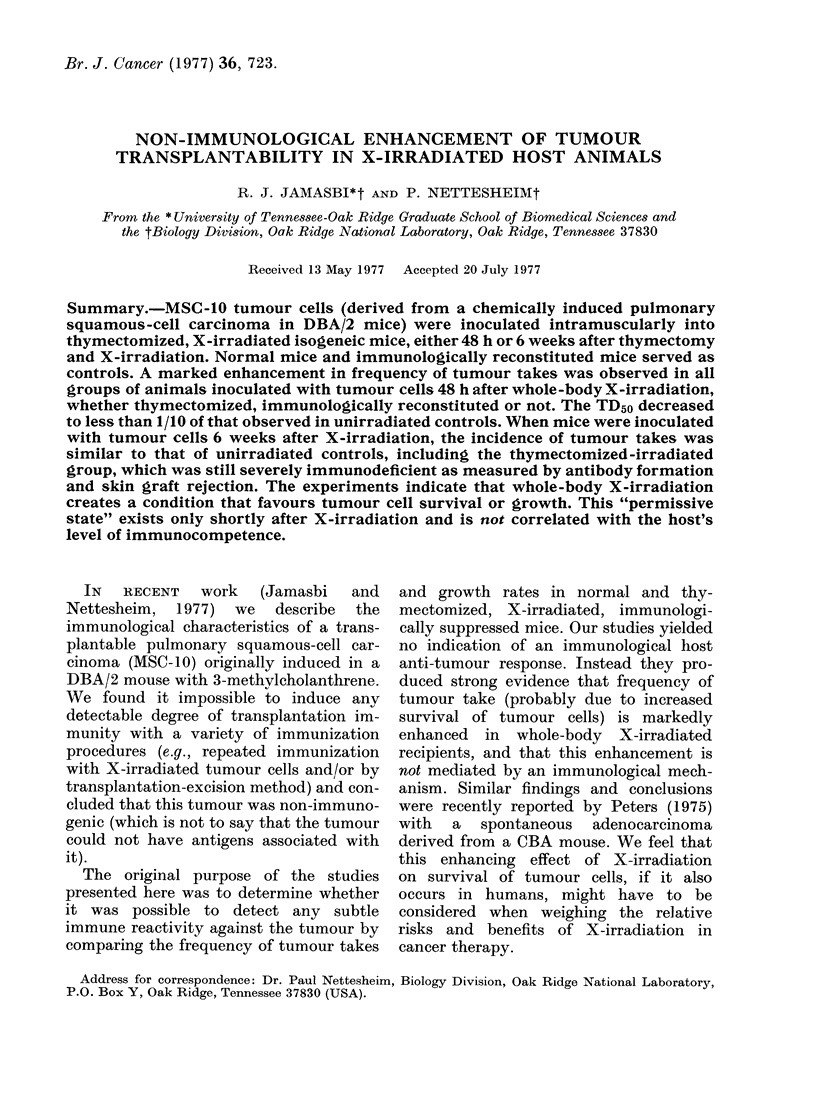

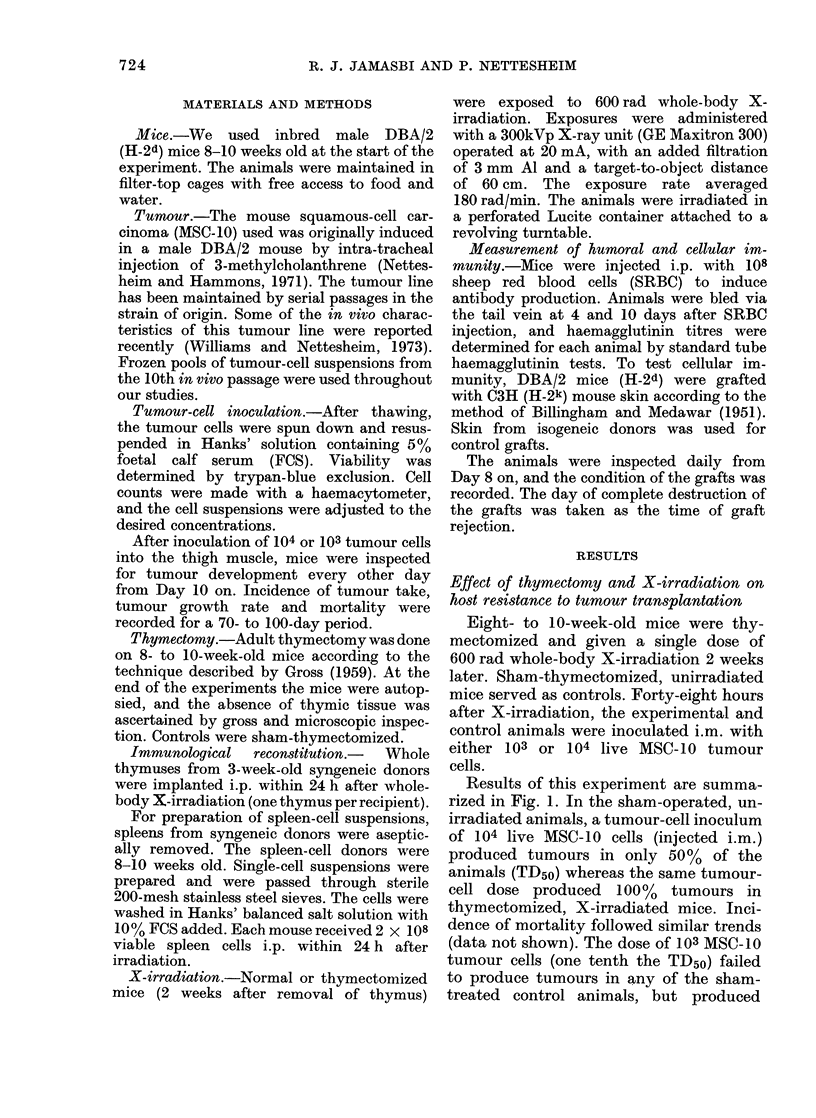

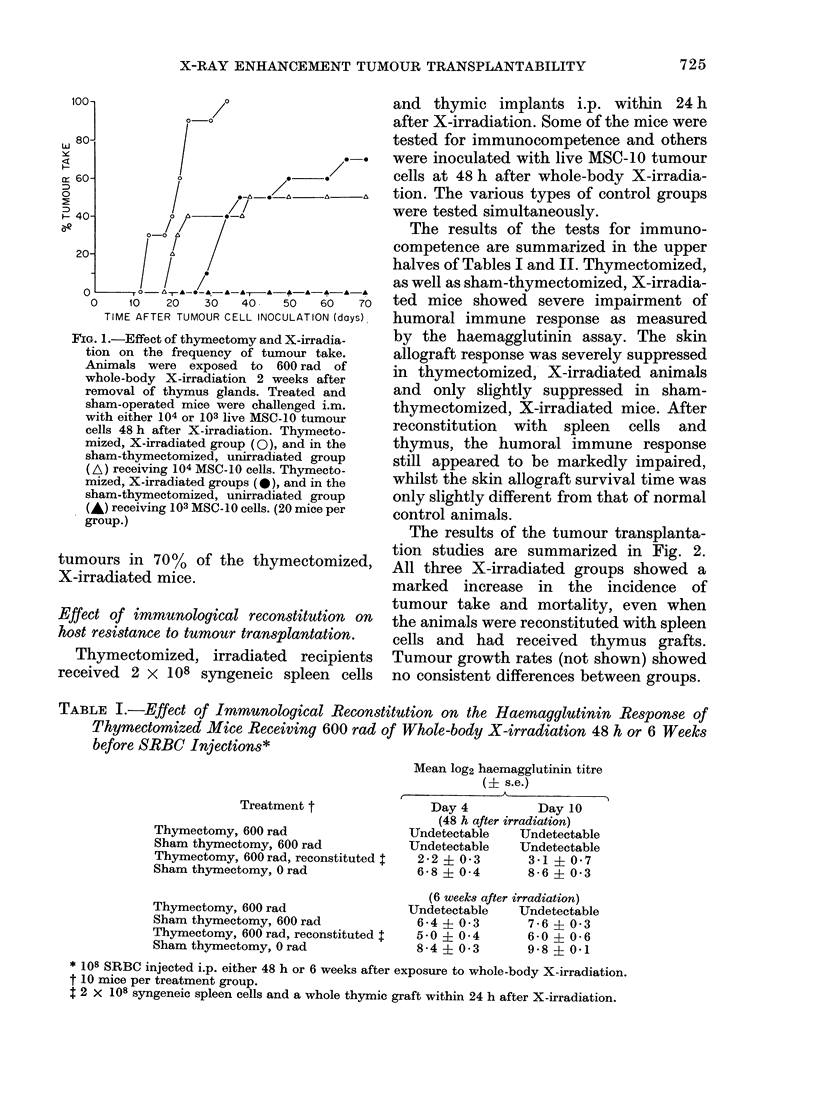

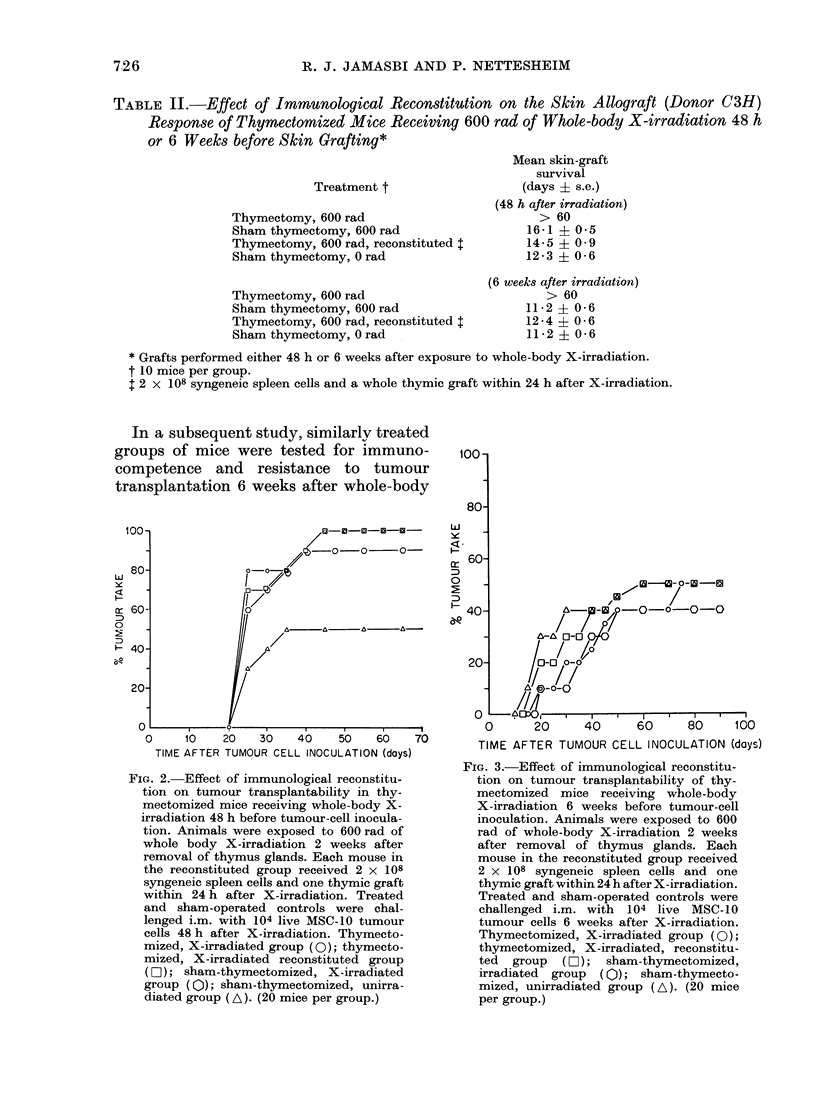

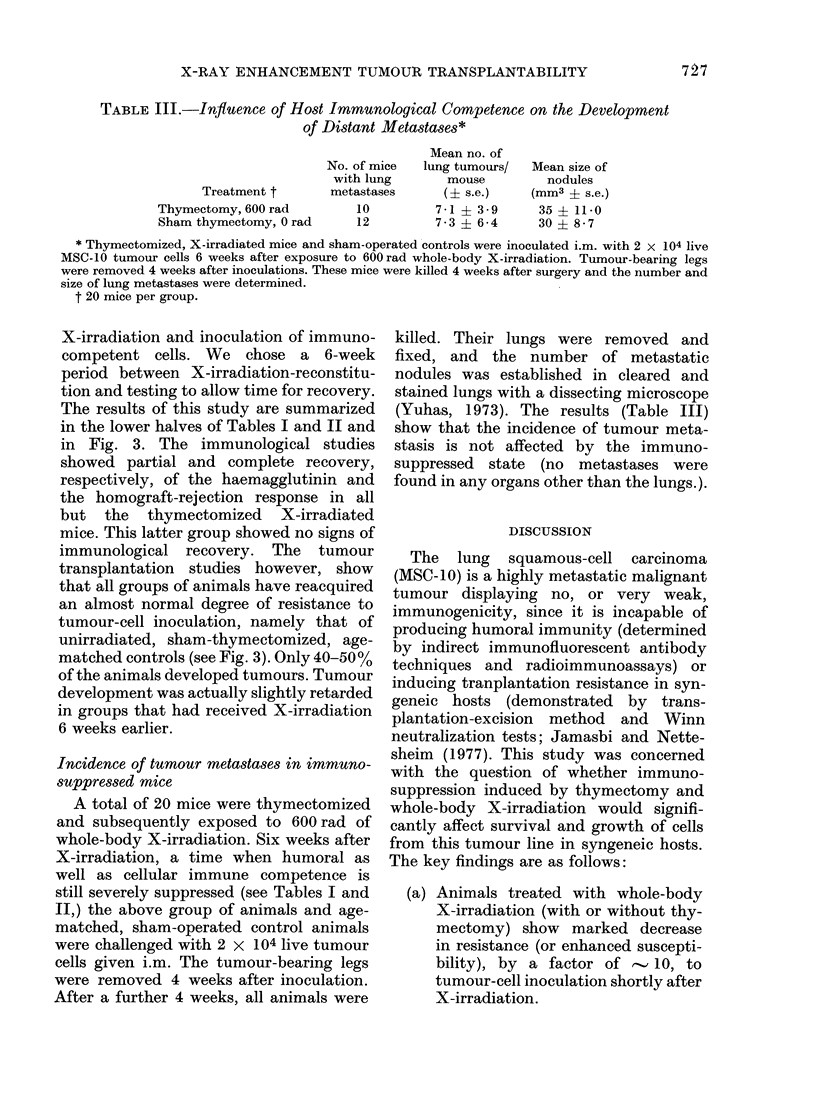

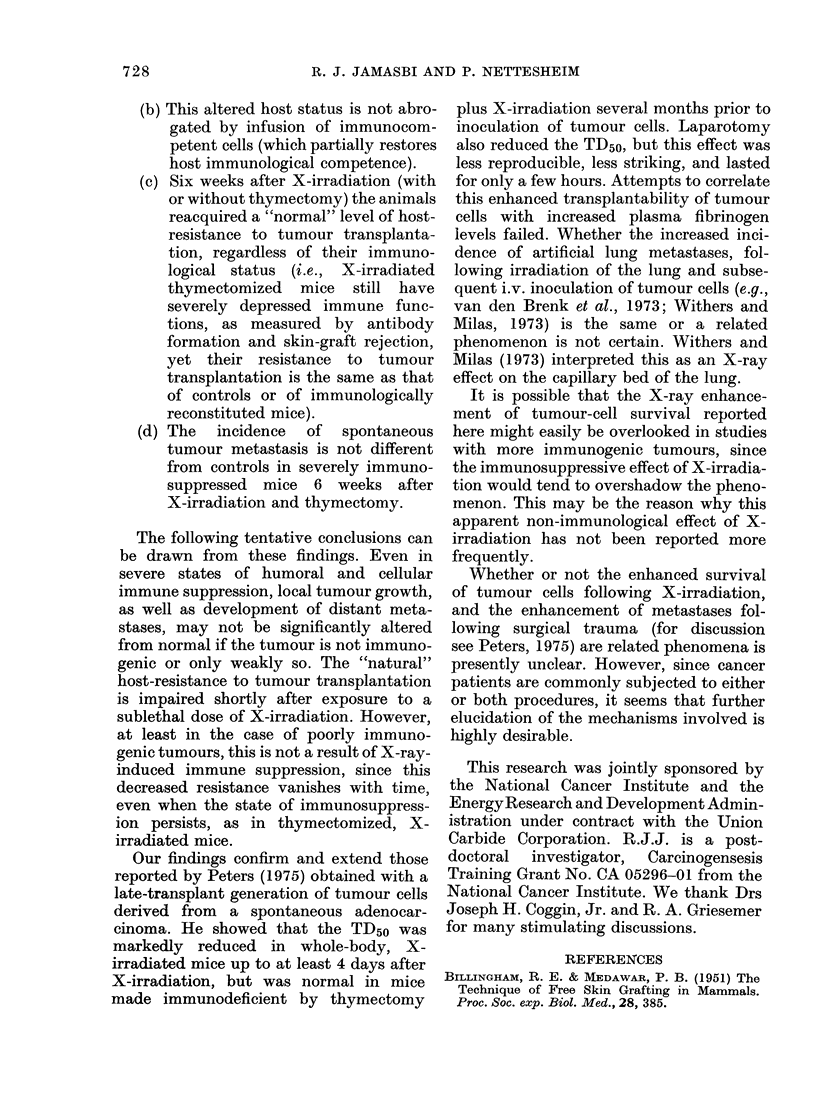

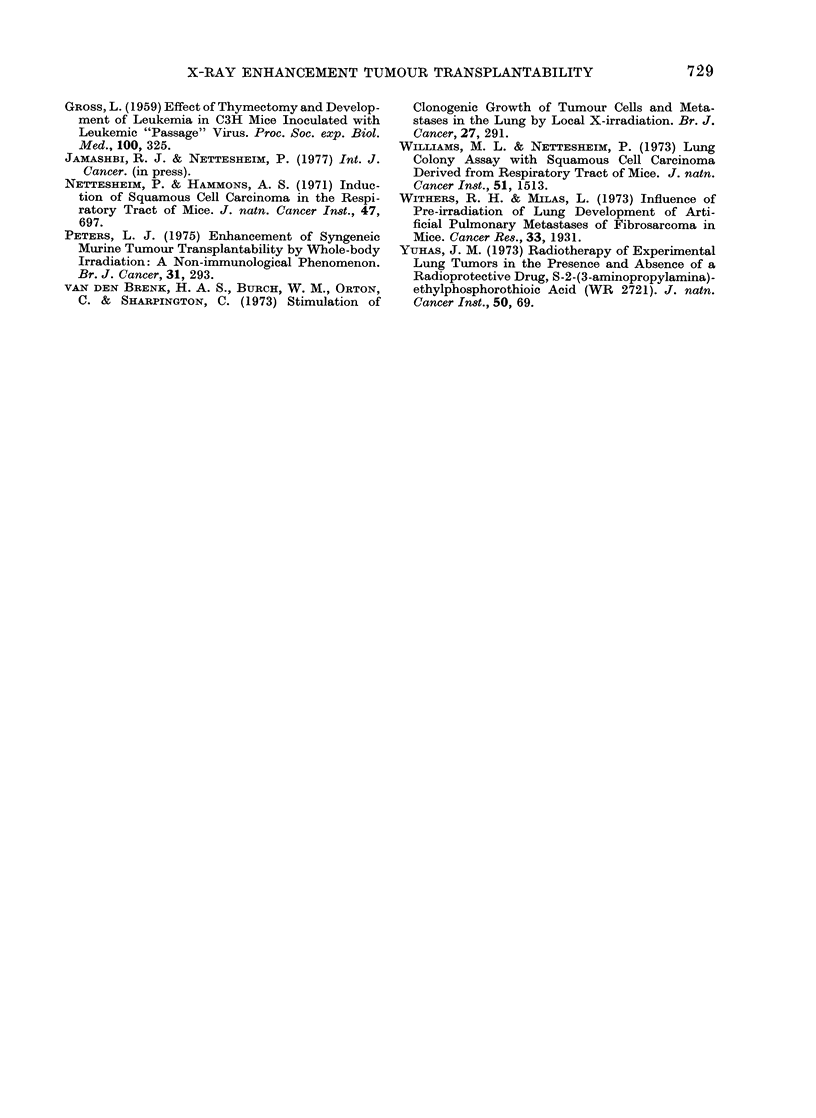


## References

[OCR_00709] GROSS L. (1959). Effect of thymectomy on development of leukemia in C3H mice inoculated with leukemic passage virus.. Proc Soc Exp Biol Med.

[OCR_00719] Nettesheim P., Hammons A. S. (1971). Induction of squamous cell carcinoma in the respiratory tract of mice.. J Natl Cancer Inst.

[OCR_00725] Peters L. J. (1975). Enhancement of syngeneic murine tumour transplantability by whole body irradiation--a non-immunological phenomenon.. Br J Cancer.

[OCR_00731] Van Den Brenk H. A., Burch W. M., Orton C., Sharpington C. (1973). Stimulation of clonogenic growth of tumour cells and metastases in the lungs by local x-radiation.. Br J Cancer.

[OCR_00739] Williams M. L., Nettesheim P. (1973). Lung colony assay with a squamous cell carcinoma derived from the respiratory tract of mice.. J Natl Cancer Inst.

[OCR_00745] Withers H. R., Milas L. (1973). Influence of preirradiation of lung on development of artificial pulmonary metastases of fibrosarcoma in mice.. Cancer Res.

[OCR_00751] Yuhas J. M. (1973). Radiotherapy of experimental lung tumors in the presence and absence of a radioprotective drug, S-2-(3-aminopropylamino)ethylphosphorothioic acid (WR-2721).. J Natl Cancer Inst.

